# Moving patient-oriented research forward: thoughts from the next generation of knowledge translation researchers

**DOI:** 10.1186/s40900-018-0110-6

**Published:** 2018-08-01

**Authors:** Andrea C. Bishop, Meghan J. Elliott, Christine Cassidy

**Affiliations:** 1 Strengthening Transitions in Pediatric Care, IWK Health Centre, 5850/5980 University Avenue, Halifax, NS B3K 6R8 Canada; 2 Department of Medicine, University of Calgary, 1403 29 Street NW, Calgary, AB T2N 2T9 Canada; 3 School of Nursing, Dalhousie University, 5896 University Avenue, Halifax, NS B3H 4R2 Canada

**Keywords:** Patient-oriented research, Patient engagement, Knowledge translation

## Abstract

**Plain English summary:**

As knowledge translation trainee participants, we report on the discussions that took place during the 2017 Knowledge Translation Canada Summer Institute. The theme of the institute was patient-oriented research and patient engagement in research. Trying to move knowledge into health care practice can be difficult. Including patients and families as members of the research team can help to overcome some of these challenges by producing more relevant research designs and results. However, in the absence of guidelines and best practices, it can be difficult for trainees and researchers to effectively engage patients and families in designing and conducting research. We detail how trainees and early career researchers are currently engaging patients in their research, the strengths and challenges of engaging patients in research, and lessons learned. These discussions have helped us to identify important areas where future training and guidance is needed to support trainees as patient-oriented researchers.

**Abstract:**

**Background**

Moving knowledge into health care practice can present a number of challenges for researchers. Including patients and families as members of the research team can help to overcome some of these challenges by producing more relevant research designs and results. However, many trainees and researchers experience difficulty in engaging patients and families in research effectively.

**Main body**

We report on the discussions that took place at the 2017 Knowledge Translation (KT) Canada Summer Institute (KTCSI). The theme of the KTCSI was patient-oriented research and patient engagement in research. We provide an important viewpoint on how trainees and early career researchers are currently engaging patients in their research, the strengths and challenges of engaging patients in research, and lessons learned. As the target audience of the KTCSI, we provide our thoughts on what is needed to support trainees and researchers to more effectively engage patients and families in research.

**Conclusion**

While many of the participants at the KTCSI are conducting patient-oriented research, practical guidance, resources and tools are needed to ensure the effective engagement of patients in research. These discussions have helped us to identify how to move forward as patient-oriented researchers and where future work and support is needed to achieve effective engagement.

## Background

With the understanding that research findings often fail to change clinical and health system practice, [1] knowledge translation (KT) science has become a growing field aimed at improving the relevance of research and uptake of its findings in the health care system [2]. The Canadian Institutes for Health Research (CIHR) defines KT as “a dynamic and iterative process that includes the synthesis, dissemination, exchange and ethically sound application of knowledge to improve the health of Canadians, provide more effective health services and products, and strengthen the healthcare system [3].” In an effort to pool KT knowledge, expertise, and resources, a network of Canadian KT experts established KT Canada in 2009 to provide ongoing education, training, and support to facilitate and advance the use of evidence in health care. Each year, the KT Canada training committee identifies a priority topic area to explore during the KT Canada Summer Institute (KTCSI), an annual intensive workshop aimed at building KT skills and networking capacity for KT research trainees and early career investigators [4]. In response to the CIHR Strategy for Patient-Oriented Research (SPOR) [5] and the demand for capacity-building in this area, the topic area chosen for the 2017 KTCSI was patient-oriented research. Patient-oriented research refers to “a continuum of research that engages patients as partners, focuses on patient-identified priorities and improves patient outcomes. This research, conducted by multidisciplinary teams in partnership with relevant stakeholders, aims to apply the knowledge generated to improve healthcare systems and practices [5].” The tenets of patient-oriented research are in direct alignment with the principles of integrated knowledge translation (iKT), in which researchers and stakeholders engage in a collaborative model of research to enhance the relevance of their findings [6].

Attendance at the KTCSI is competitive – 45 participants submitted applications in 2017, and capacity was capped at 36 participants to facilitate interactivity in sessions. Participants represented 17 unique institutions in Canada and 3 institutions in the United States, including universities, government, hospital research institutes, and funding bodies. A range of university departments were represented, including medicine, nursing, health behaviour, health sciences, psychology, human development, biomedical sciences, and rehabilitation sciences. A total of 15 faculty and facilitators, including two patient advisors, participated in the KTCSI and brought a diverse range of expertise and experience with patient-oriented research. The two patient advisors represented independent patient advisory networks and were active in their respective provincial SPOR units. They were present throughout the KTCSI, co-presented with other faculty members and offered mentoring sessions to participants.

This paper presents an overview of the 2017 KTCSI, including patient engagement activities, lessons learned, and future directions for the next generation of patient-oriented researchers. In this article, we provide our trainees’ perspective of the KTCSI and its relevance to the conduct of patient-oriented research by post-graduate trainees and early career investigators. We hope this provides readers with important insight into one of the many perspectives on patient engagement in research and helps to further the dialogue regarding training capacity in this area.

## Methods

As a junior facilitator at the KTCSI, AB was responsible for co-leading interactive sessions which sought real-time feedback from participants in targeted areas of patient-oriented research. This included identifying stakeholders with whom they were working, stages of research in which they had involved patients, the extent of patient engagement in their research, and their perceived strengths and challenges related to patient engagement in research. Poll Everywhere© software was used to facilitate report-back and consolidate findings. Findings were analyzed using descriptive statistics provided within the Poll Everywhere© platform, which was also used to generate word clouds of nominal data provided by participants. Word clouds provide a visualization of words reported, with words that are most frequently reported being biggest in size. Participants also completed final evaluations of the KTCSI where they were asked to rank each session (speaker, format, content) on a five-point scale from poor to excellent and provide additional written information regarding the most and least useful sessions, relevance of the KTCSI, and other general comments. The next section will present findings from participant report-back sessions and final evaluations.

### Findings from interactive participant sessions

#### Who should be engaged?

Participants identified a number of stakeholders that they felt were important to have on their research teams to support patient-oriented research. The majority of the discussion centered on participant confusion regarding involving patients as the targets of research versus engaging patients as members of the research team. The lines between involvement in the research and engagement in the research process were felt to be nebulous, and this discussion proved to be an important springboard over the course of the KTCSI. Not surprisingly, many participants (22%) saw opportunities to more fully engage patients during all stages of their research to become truly patient oriented. Additionally, a number of other stakeholders were seen as important to ensure that research evidence was effectively translated into patient care, including health care providers, family members, decision-makers, community groups, other researchers, health care organizations and funding agencies.

#### What are the strengths of engaging patients in research?

**Fig. 1:**
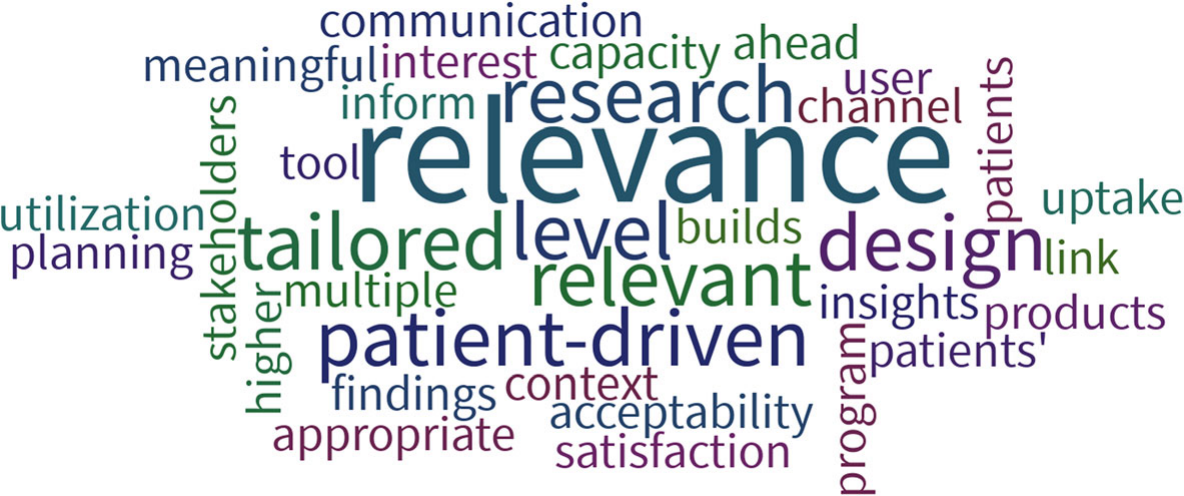
Participant identified advantages of engaging patients in research

Participants were asked to list and discuss what they perceived as the strengths of engaging patients in their work, which were discussed further in small groups. Participants put forth a number of strengths and opportunities they felt contributed to effective engagement (Fig. 1).

Improved relevance of both the research being conducted and its findings were overwhelmingly seen as the greatest strengths of engaging patients. Similarly, participants saw the value in producing research outputs that are “designed by them, for them” and are tailored to the needs of the end-users. While other studies have identified improved recruitment and retention as an important strength of engagement, participants did not identify this as an important advantage [7]. Participants also saw the importance of improving patient-oriented research capacity – both for the researchers who are engaging patients and the patients who are being engaged on the research team. The opportunity to share knowledge and expertise from the perspective of a researcher and as a patient was seen as beneficial to improve the overall research experience [8].

#### What are the challenges of engaging patients in research?

**Fig. 2:**
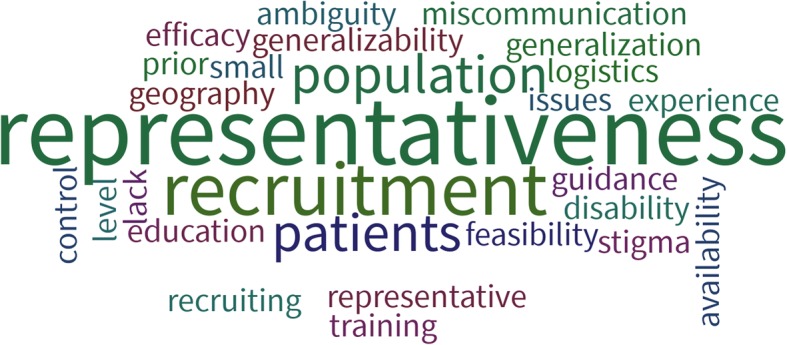
Participant identified challenges of engaging patients in research

Participants identified a number of potential challenges to engaging with patients as partners in research. This included challenges associated with identification and recruitment of patients as research team members (Fig. 2).

Perceived challenges included finding representative patients, locating patients when the population or geographic size might be small, and targeting patients and families that are from hard-to-reach socioeconomic segments. Participants struggled with the logistics and feasibility of keeping patients engaged throughout a research project, as well as how to address issues such as overcoming stigma for vulnerable populations and potential miscommunication between researchers and patients. Overall, the lack of guidance, resources and training on achieving effective patient engagement in research was seen as a significant challenge that needs to be addressed as we move forward with our research careers [9].

#### At what stages of research are you currently engaging patients?

With the understanding that patient-oriented research seeks to involve patients in all aspects of the research process, from identifying research questions to interpreting and disseminating research findings, we sought to explore how participants were engaging patients in their work. There were high levels of patient engagement in selecting the research design (16%), conducting the research (32%), and interpreting the results (24%). However, very few were engaging patients in defining the research problem (12%), formulating hypotheses (8%), reporting study findings (12%) and disseminating research findings (4%). No participants had involved patients in literature review/synthesis work. These findings align with existing evidence indicating that most research engages patients during the preparation and early stages of execution of a research study (e.g., study design, recruitment), and less commonly during data collection, analysis and translation [7].

#### To what degree are you engaging patients in research?

Finally, participants were asked to report on the extent to which they were engaging patients in their research, referring to the International Association for Public Participation (IAP2) Public Participation Spectrum [10]. This spectrum outlines levels at which patients and the public can engage in research, from low-level (e.g. keeping participants informed) to high-level (e.g. patient-initiated and patient-led research) engagement. Interestingly, levels of engagement varied, with most currently engaging patients at the “involve” level (38%), by working directly with patients throughout their research, and at the “collaborate” level (33%), through true partnership with patients to produce research findings. By comparison, the engagement literature suggests that patient and public involvement in health research and policy development is concentrated at the “consult” and “involve” levels of the spectrum [11]. A few participants identified their engagement work at the “inform” and “consult” levels, which were felt to be congruent with the types of research they were conducting. To date, none of the participants had engaged with patients at the “empower” level, whereby patients lead the research and assume responsibility for final decisions.

### Lessons Learned & Moving Forward

Three key learnings emerged at the conclusion of the 2017 KTCSI, which offer a direction for future work and guidance in this field. Firstly, we noted confusion from KTSCI trainees around the concepts of patient-oriented research and patient engagement and the traditional roles that patients play as targets and participants of health research. Participants indicated on their final evaluations that discussions regarding what patient engagement encompasses and the science versus the practice of patient engagement were the most applicable and useful for their current research. The session highlighting the differences and similarities between patient-oriented research and the concept of patient engagement elicited important discussion. Patient-oriented research, while encompassing and championing patient engagement throughout the research, also incorporates concepts related to broader stakeholder input, multidisciplinary research and application to practice [5]. Others have suggested that patient-oriented research is the intersection of patient engagement and knowledge translation [12]. By the end of the KTCSI, there remained lingering questions regarding what constitutes patient engagement. For example, does a focus group eliciting patient feedback on a proposed intervention constitute patient engagement? What if it leads to prioritizing the next research question? What is the difference between patients participating in research and patients partnering in the research process? Many approaches to research that purport to engage the community use the language of patient involvement and engagement, but do not meaningfully involve them in the research process. This could include, for example, capturing the patient voice using qualitative methods (such as focus groups), which then informs the research findings rather than subsequent study design and conduct. It is clear that this has created a great deal of confusion for trainees.

As such, greater standardization of terminology in training programs to uphold a robust definition of patient engagement as partners throughout the research process, rather than participants in research, is needed. We recommend that health professional schools adopt the definition put forth by the CIHR that upholds that patients actively participate in the identification of research priorities and questions and in the design and undertaking of research projects [13]. Further, participants expressed concern that patient engagement may become a catch-all term, or buzzword, under which all patient-related research falls, regardless of the type and extent of engagement. This is partly due to the rise of granting competitions under the SPOR umbrella and internationally, which require patient engagement throughout the research process. Without clear guidance, including resources and tools to support this work, effective engagement could be compromised. Continuing these conversations is important, and while it may never be black and white, defining clear boundaries is necessary to support KT trainees and early career researchers moving forward.

Secondly, challenges related to the recruitment and retention of patients and families, as well as decision-makers, as partners in research led to a broader discussion of the importance of building and sustaining relationships with stakeholders as we develop our research programs [8]. Many faculty and facilitators commented on the importance of having reciprocal relationships in place prior to funding calls and the important role relationship building has on engagement. Although navigating these relationships can be difficult, it is encouraging to see so many trainees and early investigators doing it as “business as usual.” While senior researchers may view this as a new way of doing things, many of us at the beginning of our careers have been trained in environments where patient and stakeholder engagement is fundamental to the research we do. That being said, we discussed significant challenges in recruiting, establishing, and maintaining research partnerships among diverse patient and family stakeholders, and vulnerable populations, in particular. Although optimal engagement strategies in such populations remain unclear, acknowledgement of this concern highlights the careful consideration participants are giving to patient partner identification and the implications of the resulting research. To help address this challenge, we recommend trainees utilize established patient networks, such as Patient Advisors Network and Patient Voices Network, to engage patients before they begin their research projects. These networks follow an established process to identify and link patient partners and researchers, as well as providing important guidance on how to sustain and strengthen engagement throughout the research process. This approach not only reduces the onus on trainees to establish relationships on their own, but also contributes to the establishment of the patient engagement community throughout Canada.

Many participants also recognized the tendency to engage ‘professional patients’ (e.g., those who may be patient advisors on a number of research projects) and identified the need for greater efforts to partner with patients and families from underrepresented populations in patient-oriented research. These may include individuals who are stigmatized by their health condition, the elderly, persons with chronic conditions, those from lower socioeconomic areas, those who speak English as a second language, and visible minorities, to name a few. When we fail to include representative patients, not only are we failing to identify important research priorities, but our research results and KT products will also fail to reach and impact these populations. It is imperative that trainees and early career researchers endeavor to recruit more inclusive voices to add to our current dialogue and to establish effective ways of inviting these populations to the table. Although the KTCSI included two patient advisors throughout the program, participants suggested that greater patient and stakeholder presence, as well as informal opportunities to interact, would be helpful for future patient engagement sessions. Finally, although SPOR has highlighted inclusiveness as a guiding tenet for conducting and evaluating patient-oriented research, we encourage trainees and researchers to evaluate and share their methods for recruiting underrepresented populations to help inform best practices in this area.

Finally, trainees and early career researchers perceived a critical gap in the availability of resources and guidance on how to engage patients in research. Participant evaluation comments indicated that many are now considering new ways to include patients during all stages of the research process; however, uncertainty regarding practical ways to do this remained. For example, how does one find representative patients? How can we invest in capacity building? What type of remuneration should be offered? Is it always appropriate to engage patients throughout the research process? When is engagement a burden? Current dialogue on patient engagement in research suggests that patients should be engaged in all stages in the design and conduct of research, and in effect become co-researchers. However, this poses significant challenges for both patients and research teams, including protracted timelines, financial and human resources, and potential conflicts between desired patient and research outcomes. Trainees and early career researchers at the KTCSI identified many challenges with engaging patients, such as the feasibility and logistics of engagement, as well as compensation for patient’s time, and these cannot be overlooked as important areas of evidence building and syntheses. A lack of best practice guidance on conducting patient-oriented research has been reported in the literature, [7] as has a lack of practical tools for engaging patients and families [9]. In the absence of such tools, we recommend following the guidance of the Institute for Patient and Family Centered Care (IPFCC) in creating open and honest communication between trainees, researchers and patients engaged in their work regarding what level of engagement is appropriate for each patient and each research project [14]. Use of the IAP2 framework can help shape these discussions and provide an objective and standardized way to define how patients were engaged [10]. Improved evaluation of patient engagement strategies and outcomes was also identified by participants as an important area for future research and to establish an important evidence base to support KT science [15].

## Conclusions

The next generation of KT researchers considers engaging in patient-oriented research as a means to advance the translation of research findings into practice. While many of the participants at the KTCSI are conducting patient-oriented research, practical guidance, resources, and tools are needed to ensure the effective engagement of patients in research. Many of the issues identified by KTCSI participants were not KT specific, but rather speak to the need for more universal guidance on engaging patients in research. It is, however, important for KT trainees and early career researchers to be at the forefront of evidence creation and synthesis in this field. This will help advance the science of iKT and ensure that KT products remain relevant and responsive to the needs of patients and other end-users.

The KTCSI provides an important venue for trainees, early career researchers, and KT Canada faculty members to come together and discuss important issues such as patient engagement. We feel it is important to highlight the experiences and challenges faced by trainees and early career researchers as actors in shaping the future of patient engagement in research. The opportunity afforded by the KTCSI for novice researchers to share their experiences with patient engagement and contribute to best practices supports future collaborations between patients and the research community. We encourage KT Canada faculty to continue these discussions with their trainees and uphold patient engagement as a priority area. We also encourage KT trainees to continue to share their learnings with one another through participation in events like the KTCSI and through knowledge exchange opportunities like the KT Canada Seminar Series. Finally, we encourage SPOR support units across Canada to provide ongoing training and guidance regarding patient-oriented research, and specifically patient engagement, to trainees through workshops and patient advisor mentoring, and to build capacity in this area by providing targeted trainee funding opportunities to conduct this work. Having these support structures in place will help trainees to incorporate, share, and grow engagement best practices in their current and future work. Establishing and meeting trainee needs to conduct high quality and rigorous patient oriented research will, in turn, contribute to the advancement of patient engagement science.

## Abbreviations

CIHR: Canadian Institute of Health Research
IAP2: International Association for Public Participation
KT: Knowledge Translation
KTCSI: KT Canada Summer Institute

## References

[1] Grimshaw JM, Eccles MP, Lavis JN, Hill SJ, Squires JE (2012). Knowledge translation of research findings. Implement Sci.

[2] Straus SE, Tetroe JM, Graham ID (2011). Knowledge translation is the use of knowledge in health care decision making. J Clin Epidemiol.

[3] Canadian Institutes of Health Research (2012). Guide to Knowledge Translation Planning at CIHR: Integrated and End-of-Grant Approaches - CIHR.

[4] Straus SE, Brouwers M, Johnson D, Lavis JN, Légaré F, Majumdar SR (2011). Core competencies in the science and practice of knowledge translation: description of a Canadian strategic training initiative. Implement Sci.

[5] Canadian Institutes of Health Research (2010). Strategy for Patient-Oriented Research.

[6] Gagliardi AR, Berta W, Kothari A, Boyko J, Urquhart R (2016). Integrated knowledge translation (IKT) in health care: a scoping review. Implement Sci.

[7] Domecq JP, Prutsky G, Elraiyah T, Wang Z, Nabhan M, Shippee N (2014). Patient engagement in research: a systematic review. BMC Health Serv Res.

[8] Shippee ND, Domecq Garces JP, Prutsky Lopez GJ, Wang Z, Elraiyah TA, Nabhan M (2015). Patient and service user engagement in research: a systematic review and synthesized framework. Health Expect.

[9] Kovacs Burns K, Bellows M, Eigenseher C, Gallivan J (2014). “Practical” resources to support patient and family engagement in healthcare decisions: a scoping review. BMC Health Serv Res.

[10] International Association for Public Participation. IAP2's Public Participation Spectrum. https://cdn.ymaws.com/www.iap2.org/resource/resmgr/foundations_course/IAP2_P2_Spectrum_FINAL.pdf. Accessed 9 July 2018.

[11] Ocloo J, Matthews R. From tokenism to empowerment: progressing patient and public involvement in healthcare improvement. BMJ Qual Saf. 2016. 10.1136/bmjqs-2015-004839. 10.1136/bmjqs-2015-004839 PMC4975844 26993640

[12] McGavin C (2017). Patient-oriented research.

[13] Canadian Institutes of Health Research. Patient engagement. 2012. http://www.cihr-irsc.gc.ca/e/45851.html. Accessed 27 June 2018.

[14] Institute for Patient and Family-Centered Care. Nurturing and Sustaining Partnerships Tools and Resources. http://www.ipfcc.org/bestpractices/sustainable-partnerships/tools/nurturing.html. Accessed 25 Aug 2017.

[15] Esmail L, Moore E, Rein A (2015). Evaluating patient and stakeholder engagement in research: moving from theory to practice. J Comp Eff Res.

